# A retrospective analysis on metastatic rate of the internal mammary lymph node and its clinical significance in adjuvant radiotherapy of breast cancer patients

**DOI:** 10.1186/s12885-020-6642-9

**Published:** 2020-02-24

**Authors:** Li Li, Hongyan Zhang, Linwei Wang, Conghua Xie, Yunfeng Zhou, Yahua Zhong

**Affiliations:** 1 Department of Radiation and Medical Oncology, Zhongnan Hospital of Wuhan University, Wuhan, China; 2 Hubei Cancer Clinical Study Center, Wuhan, China; 3 Hubei Key Laboratory of Tumor Biological Behaviors, Wuhan, China

**Keywords:** Breast cancer, The internal mammary lymph node, Metastasis, Radiotherapy

## Abstract

**Background:**

There is a discrepancy about the metastatic rate of internal mammary lymph nodes (IMNs) between clinical and pathologic findings. We aimed to investigate the metastatic rate of IMNs and to provide recommendations on target volume delineation of IMNs for adjuvant radiotherapy in breast cancer patients.

**Methods:**

We retrospectively analyzed data from 114 breast cancer patients treated with surgery without adjuvant radiotherapy who developed local and/or regional lymph node recurrence/metastasis at our institute from January 2015 to January 2019. Patients with widely lung or pleural metastases were excluded. We first analyzed the recurrence rate with the chest wall, the metastatic rate of internal mammary/anterior mediastinal, ipsilateral axillary and supraclavicular lymph nodes, and then investigated the distribution of the IMNs.

**Results:**

Among the 114 included patients, the recurrence rate with the chest wall, metastatic rate of IMNs, IMNs/anterior mediastinal lymph nodes, ipsilateral axillary lymph nodes, and the ipsilateral supraclavicular lymph nodes was 43, 37.7, 59.6, 12.3, and 22.8%, respectively. The metastatic IMNs were mainly located from the first to the second intercostal space. However, metastatic lymph nodes could also be observed above the upper edge of the first rib.

**Conclusions:**

The metastatic rate is high in the IMNs and irradiation of the internal mammary lymphatic chain is required. It is suggested that the upper bound of the internal mammary lymphatic chain should be up to the subclavian vein with a 5-mm margin, thus connecting to the caudal border of supraclavicular clinical target volume in breast cancer patients at high risk of recurrence.

## Background

Breast cancer is the most common malignant tumor in women, and postoperative adjuvant radiotherapy is an important mode of treatment [1, 2]. Based on results from two clinical trials (MA.20 and EORTC 22922/10925) [3, 4], the 2016 National Comprehensive Cancer Network (NCCN) guidelines strongly recommend irradiation of internal mammary lymph nodes (IMNs) in patients with 1–3 positive axillary lymph nodes (ALNs) (category 2A), following mastectomy and lumpectomy [5]. This approach is recommended in addition to irradiation of the chest wall and supraclavicular lymph nodes in postoperative adjuvant radiotherapy.

Nevertheless, controversies persist regarding the recommendation of IMNs irradiation in all patients with ALNs metastases [6, 7]. Opponents believe that the recurrence rate of IMNs is low, within 2–5%, while irradiation of IMNs increases cardiac and pulmonary toxicity. Although the internal mammary lymphatic chain is the first-echelon nodal drainage site in breast cancer [8, 9], along with the axilla, the importance of its treatment has long been debated. The metastatic rate of IMNs was 33% after the extended mastectomy in breast cancer patients treated in the 1970s [10, 11], suggesting a discrepancy between clinical and pathologic findings. To examine this contradiction, we retrospectively analyzed data from 114 breast cancer patients treated only by surgery without adjuvant radiotherapy who developed local recurrence and regional lymph node metastases, while receiving care at our institute from January 2015 to January 2019. We aimed to investigate metastatic rate and provide recommendations on target volume delineation of IMNs for adjuvant radiotherapy in surgically treated breast cancer patients.

## Methods

### Patient selection

**Fig. 1:**
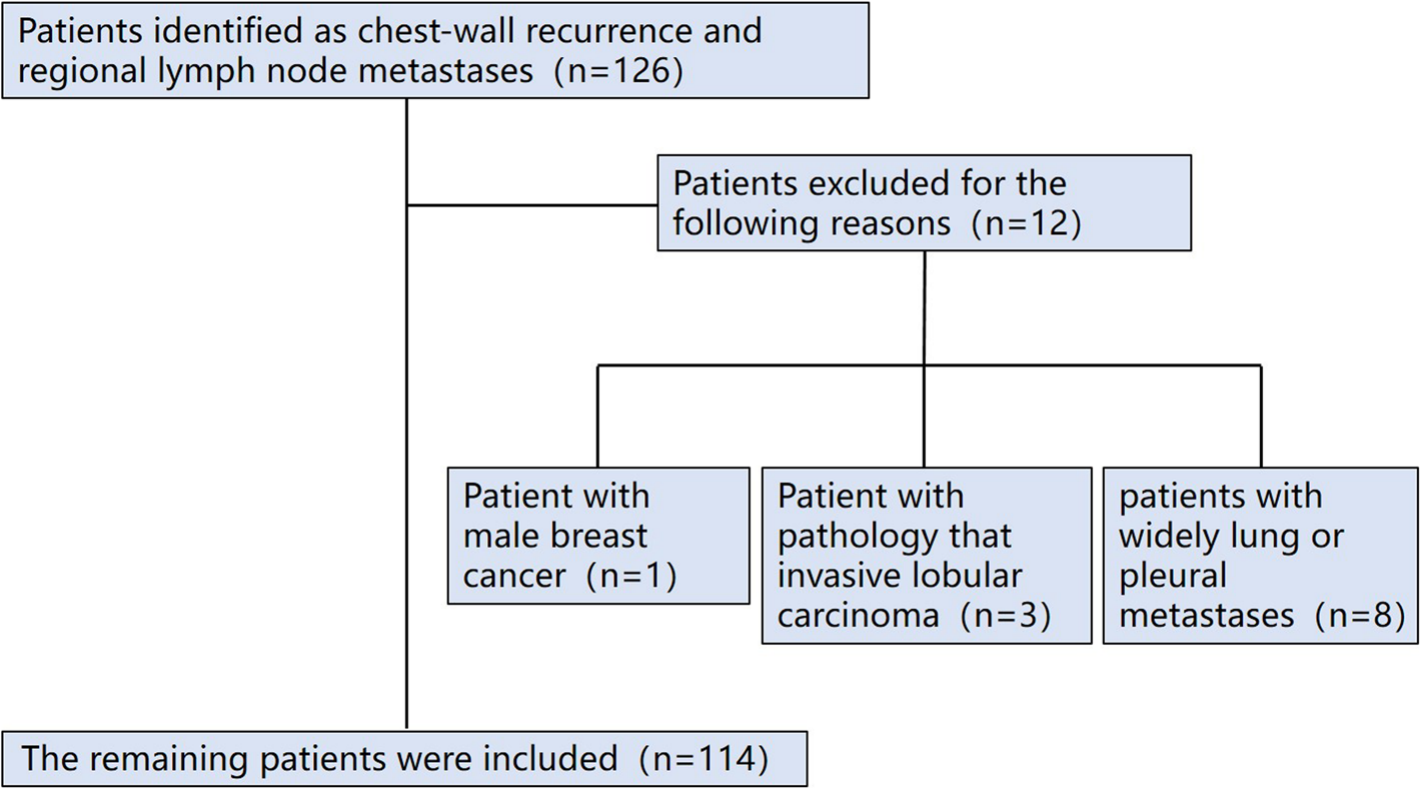
Flowchart representing selection process of patients included in this study

This is the selection process of patients included in this study (Fig. 1): 126 patients were initially identified as having chest-wall recurrence and regional lymph node metastases from January 2015 to January 2019. Twelve patients were subsequently excluded for the following reasons: 1 patient was excluded with the reason that he was male breast cancer patient; 3 patients were excluded with the reason that the pathology was invasive lobular carcinoma; 8 patients had widely lung or pleural metastases, and therefore not included. The remaining 114 patients were included in this study.

### Diagnostic criteria

Eligibility criteria included unilateral histologically confirmed invasive breast carcinoma of stage I, II, or III with any quadrant located primary tumor, irrespective of axillary involvement, eligible patients had undergone mastectomy or breast-conserving surgery and axillary dissection; exclusion criteria included patients that male breast cancer, bilateral breast cancer, and noninvasive breast carcinoma, patients with widely lung or pleural metastases were also excluded; the definition of hormone receptor-positive is estrogen and/or progesterone receptor positive (the positive rate is equal or greater than 1%), and the definition of HER2 positive is immunohistochemistry [IHC] 3+ or IHC2+/ in situ hybridization-positive. The definition of TNM stage is according to the 7th edition of AJCC cancer staging. The diagnosis of internal mammary/anterior mediastinal lymph node metastasis was mainly based on clinical symptoms, signs, and imaging modalities (mainly enhanced computed tomography [CT] of the chest). Under normal circumstances, the short diameter of IMNs on the enhanced CT is 2–5 mm. The definition of lymph node metastasis include that the internal mammary lymph nodes whose short diameter is more than 6 mm, or the lymph node is tightly connected to the internal mammary blood vessels regardless of the short diameter, lymph node who meet one of the above conditions can be seen as metastatic lymph node; the anterior mediastinal lymph nodes whose short diameter were more than 10 mm, or the lymph node is tightly connected to the blood vessels, or lymph node with ring-enhancement in contrast-enhanced CT images also can be seen as metastatic lymph node; and the hypermetabolism in PET-CT also be diagnosed as metastases even if the short diameter was less than 6 mm or 10 mm [12].

### Statistical analysis

We first analyzed the recurrence rate with the chest wall, the metastatic rate of internal mammary/anterior mediastinal, ipsilateral axillary and supraclavicular lymph nodes, and then investigated the distribution of the IMNs. In this cross-sectional study, the interval estimation of the population rate was based on approximate normal distribution method when nP and n (1-P) were both greater than five, otherwise look-up table method was used.

## Results

### Clinical characteristics of included patients

The patients’ age at onset ranged from 31 to 77 years (median age 45 years old), and none of the included 114 patients had received postoperative adjuvant radiotherapy, patients with widely lung or pleural metastases were excluded. In the present study, most tumors (82.5%) were located at the lateral quadrant; the molecular subtype of Her2 negative (Hormone Receptor (HR)+), Her2 positive (HR+/−) and TNBC (triple negative breast cancer) are 30.7, 34.2, and 35.1% respectively; and the rate of tumor staging that under stage IIIA is 64.9% (Table 1).

**Table 1:** Clinical characteristics of all patients

	Number(n=)	Percent (%)
Gender		
female	114	100
Tumor location		
medial/central	20	17.5
lateral	94	82.5
Molecular subtype	`	
Her2 negative (HR+)	35	30.7
Her2 positive (HR+/−)	39	34.2
TNBC	40	35.1
Tumor staging		
I	16	14.0
IIA	30	26.3
IIB	28	24.6
IIIA	17	14.9
IIIB	8	7.0
IIIC	15	13.2

### Clinical characteristics of patients with chest wall recurrence and regional lymph nodes metastases

In the present study sample, there were 49 (43%) cases of chest wall recurrence, 43 (37.7%) cases of IMNs metastases only, and 68 (59.6%) cases of IMNs/anterior mediastinal lymph node metastases. There were also 14 (12.3%) cases of ipsilateral ALNs recurrence and 26 (22.8%) cases of ipsilateral supraclavicular lymph node recurrence (Table 2). Regardless of tumor location, molecular subtype, or tumor staging, the metastatic rate of IMNs/anterior mediastinal lymph nodes was higher than 40% (Table 3).

**Table 2:** Clinical characteristics of chest wall recurrence and RN metastasis

	Number (n=)	Percent (%)
Chest wall recurrence	49	43.0
IMNs metastasis	43	37.7
Anterior mediastinal lymph node metastasis	58	50.9
IMNs/anterior mediastinal lymph node metastasis	68	59.6
Ipsilateral supraclavicular lymph node recurrence	26	22.8
Ipsilateral axillary lymph node recurrence	14	12.3

*RN* Regional Nodal, *IMNs* Internal Mammary Lymph Nodes

**Table 3:** Clinical characteristics of IMNs/anterior mediastinal lymph nodes metastases

	IMNs/anterior mediastinal lymph node metastases (%)	No IMNs/anterior mediastinal lymph node metastases (%)	95%CI
Tumor location			
medial/central	14 (70%)	6 (30%)	49.92, 90.08%
lateral	52 (55.3%)	42 (44.7%)	45.27, 65.37%
Molecular subtype			
Her2 negative (HR+)	17 (48.6%)	18 (51.4%)	32.01, 65.13%
Her2 positive (HR+/−)	21 (53.8%)	18 (46.2%)	38.20, 69.49%
TNBC	28 (70%)	12 (30%)	55.80, 84.20%
Tumor staging			
I	10 (62.5%)	6 (37.5%)	38.78, 86.22%
IIA	18 (60%)	12 (40%)	42.47, 77.53%
IIB	16 (57.1%)	12 (42.9%)	38.81, 75.47%
vIIIA	7 (41.2%)	10 (58.8%)	17.78, 64.57%
IIIB	6 (75%)	2 (25%)	47.00, 100.00%
IIIC	9 (60%)	6 (40%)	35.21, 84.79%

*IMNs* Internal Mammary Lymph Nodes, *CI* Confidence Interval, *TNBC* Triple Negative Breast Cancer

### Distribution of the metastatic internal mammary/anterior mediastinal lymph nodes

**Fig. 2:**
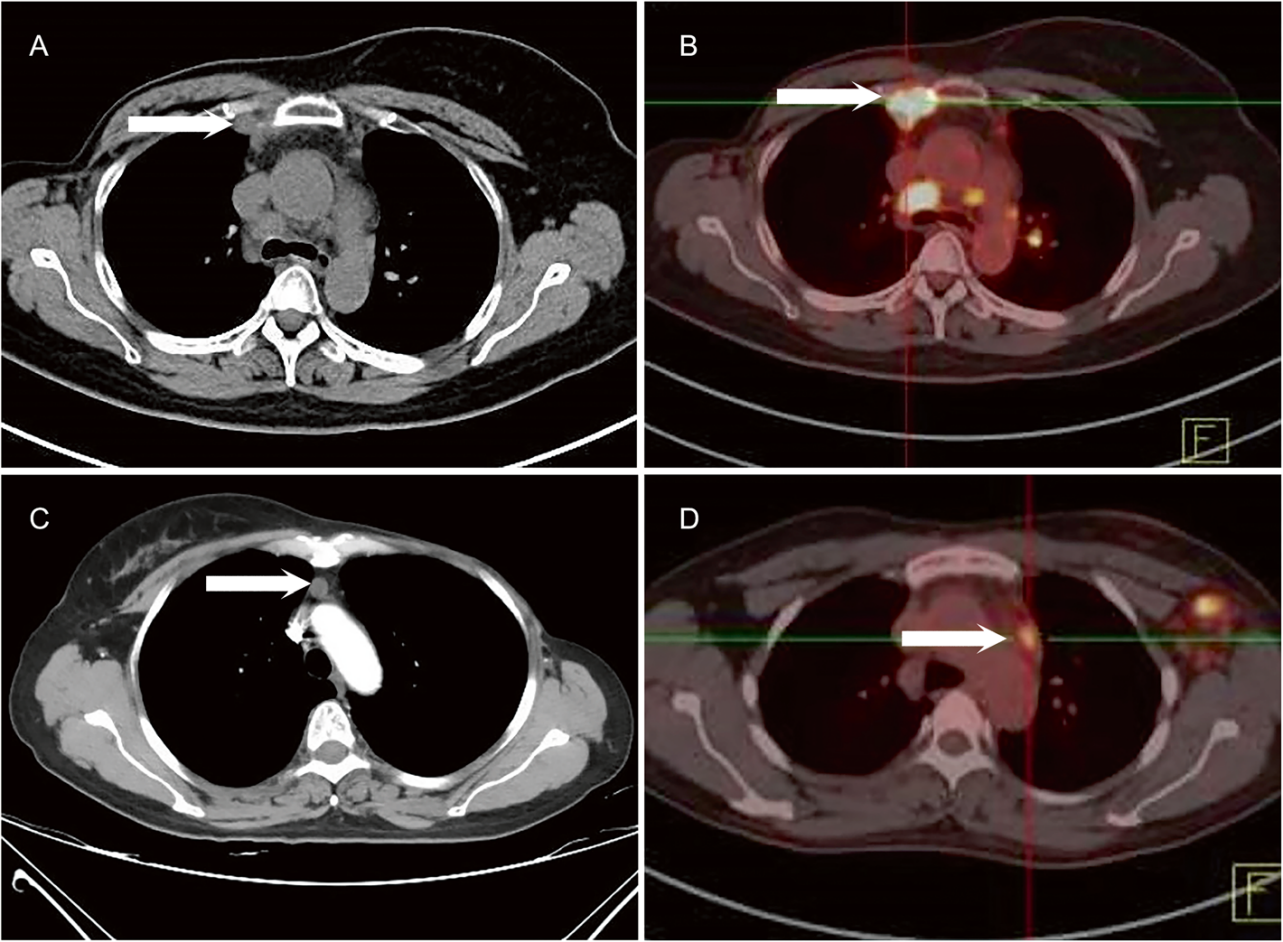
**a**-**b** metastatic internal mammary lymph nodes (white arrow); **c**-**d** metastatic anterior mediastinal lymph node (white arrow)

**Fig. 3:**
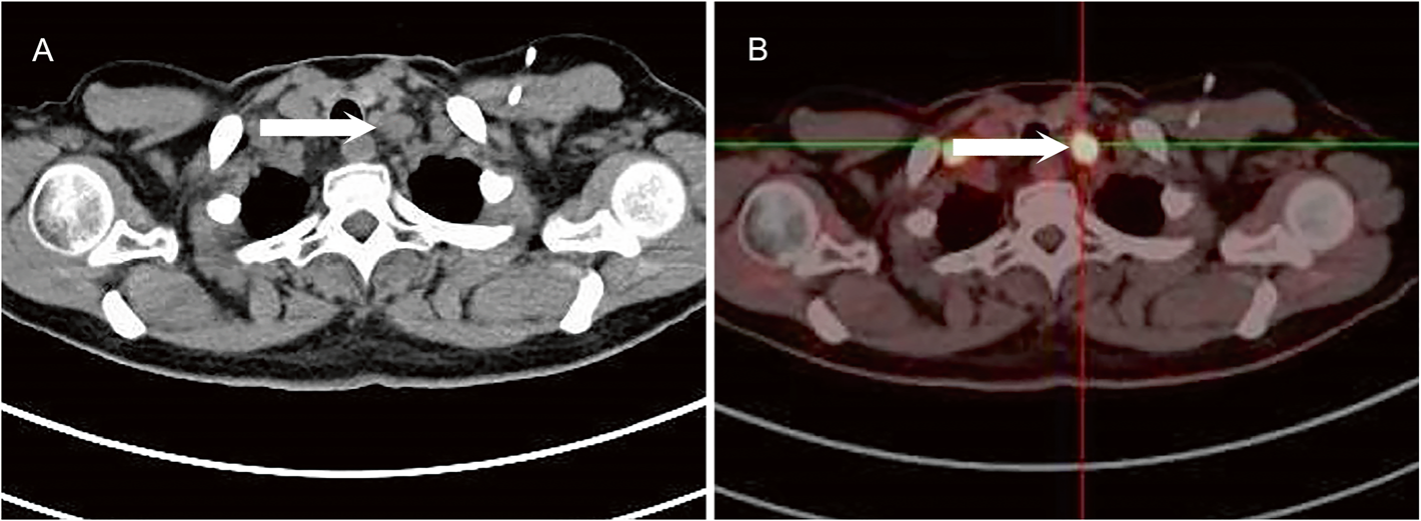
**a**-**b** metastatic lymph node above the upper edge of the first rib (white arrow)

Among the 43 patients, 45.4% of the metastatic IMNs were located at the first intercostal space, 36.4% at the second intercostal space, 9.1% at the third intercostal space, and 2.3% at the fourth intercostal space (Table 4). The IMNs were mainly located at the first to the second intercostal space (Fig. 2a-b), while the anterior mediastinal lymph nodes were mainly distributed in the region 3A and region 6 (Fig. 2c-d). Moreover, metastatic lymph nodes could also be observed above the upper edge of the first rib (Fig. 3a-b), with a metastatic rate of 7%.

**Table 4:** Anatomic distribution of IMNs metastases

Location of the metastatic IMNs	Number(n=)	Percent(%)
The upper edge of the first rib	3	7
The first intercostal space	20	45.4
The second intercostal space	16	36.4
The third intercostal space	4	9.1
The fourth intercostal space	1	2.3
The fifth intercostal space	0	0
The sixth intercostal space	0	0

*IMNs* Internal Mammary Lymph Nodes

## Discussion

The 2016 NCCN guidelines strongly recommended irradiation of the IMNs as part of the postoperative adjuvant radiotherapy for patients with 1–3 positive ALNs, following mastectomy and lumpectomy (category 2A) [5]. However, changes to the guidelines did not eliminate the controversies regarding irradiation of the internal mammary lymphatic chain. The reason was probably that the recurrence rate was only 2% according to clinical reports [13], and the irradiation dose of cardiopulmonary was increased by irradiation of the internal mammary lymphatic chain [14, 15]. In this study, we retrospectively examined data from 114 patients with local and/or regional lymph node recurrence without adjuvant radiotherapy. The recurrence rates with the chest wall, supraclavicular, and axillary lymph nodes were 43, 22.8, and 12.3%, respectively, which is consistent with the literature [16]. However, the recurrence rate of IMNs was 37.7%, second to the recurrence of the chest wall, and higher than the recurrence rate with the supraclavicular region, in the present study. So the recurrence rate of IMNs was underestimated probably. The reason might be that IMNs tend to be small, with a diameter ranging from 2 to 5 mm. As a result, routine imaging examinations easily miss IMNs, whose short diameter in excess of 6 mm qualifies as metastasis, unless a huge mass is formed at a very late stage. In addition, IMNs recurrence rarely exists in isolation, often involving systemic metastases to the liver, lung, and other organs [17], while presenting without specific symptoms, leading physicians to focus on other recurrences. In this study, the patients enrolled didn’t undergo postoperative adjuvant radiotherapy owing to various reasons such as lack of indications of radiation therapy, poor economic conditions and so on, but most of the previous studies enrolled patients that underwent postoperative radiotherapy which may reduce the metastatic rate; secondly, the reason is perhaps that nearly 30% of the patients studied underwent PET-CT examination which could improve the detection rate of metastatic lymph nodes probably; and thirdly, in our opinion, the internal mammary lymph node region and the anterior mediastinal lymph node region can be seen as an entirety (the reason could be explained below), so when the metastasis of the anterior mediastinal lymph node occures, we think that the internal mammary lymph node has a very high probability of metastasis even if the short diameter is less than 6 mm. In the present study, the recurrence rate of IMNs was very high, which is consistent with the pathologic results reported for extended mastectomy [11, 18]. It answers the clinical problem that a discrepancy between clinical and pathologic findings, which have confused us for many years, and suggests the necessity of irradiation of the internal mammary lymphatic chain. Overall, these findings indicate that IMNs should be included in the target volume, now that the supraclavicular lymph nodes need to be irradiated.

The IMNs receive lymphatic drainage from the medial and central parts of the breast and chest wall. The output tube is connected to the supraclavicular lymph nodes, leads to the anterior mediastinal lymph nodes, and enters the vein via the thoracic duct or the right lymphatic duct. Metastasis to the anterior mediastinal lymph node is not as rare as once thought. The anterior mediastinum is a stenotic region between the sternum, pericardium, and mediastinal pleura. Anatomically, the anterior mediastinal lymph nodes include the parasternal and anterior mediastinum group, while the parasternal group corresponds to the IMNs in breast cancer. The anterior mediastinum, which receives lymphatic drainage from the adjacent pleura, is not a region of high-risk lymph node metastasis even in lung cancer, except when there is pleural involvement or reflux of the enlarged mediastinal lymph node is formed. Anatomically, the anterior mediastinal metastatic lymph nodes should be derived from the internal mammary lymphatic chain, excluding widely lung or pleural metastases. Based on these considerations, we propose a concept of the extensive internal mammary lymph node region, which captures the internal mammary lymphatic chain and anterior mediastinal lymph node area (mainly region 3A and 6). While metastatic IMNs might be easily missed at routine imaging examinations, metastatic anterior mediastinal lymph nodes are larger and easier to identify on enhanced CT images therefore reducing the risk metastases being missed. Our results suggest that the metastatic rate to the anterior mediastinal lymph node is 50.9%. When the IMN and anterior mediastinum regions are combined, the recurrence rate increases to 59.6%. As such, we propose that irradiation of the internal mammary lymphatic chain is an indispensable part of adjuvant radiotherapy for breast cancer patients treated with surgery. This proposal is consistent with the NCCN guidelines [5].

Traditionally, the internal mammary lymphatic chain has been defined as the region between the first and third intercostal space [19]; however, metastatic lymph nodes could also be observed above the upper edge of the first rib. The internal mammary artery originates from the lower wall of the first segment of the subclavian artery, 1–2 cm down the lateral side of the sternum, divided into 2 branches (the superior epigastric and musculophrenic artery) until the sixth intercostal space, and accompanied by two veins. In our opinion, the internal mammary lymph node is consistent with the medial supraclavicular lymph node, once the metastases of the IMNs happened, it is easy to metastases to the medial supraclavicular region, so this is why we suggested that the upper bound of the internal mammary lymphatic chain should be up to the subclavian vein with 5 mm margin, which is connected to the caudal board of the supraclavicular CTV in breast cancer patients with high risk of recurrence.

Veronesi et al. [20] performed a follow-up study of 737 patients who had not undergone adjuvant radiotherapy and systemic therapy after radical mastectomy for 30 years. The prognosis for patients with IMNs metastases was similar to the prognosis of patients with ALNs metastases, with the 10-year disease-free survival at 59.6 and 62.4%, respectively. And both of IMNs and ALNs metastases having the worst prognosis, the 10-year disease-free survival was 37.3%. Once IMNs recurred, according to the poor prognosis of the follow-up of 6000 breast cancer patients, IMN metastasis may indicate distant metastasis [17]. In a separate study from 2009, Heuts et al. [21] reported that, during a 46-month follow-up of 764 patients, the prognosis for patients with IMN metastasis was similar to the prognosis of patients with ALN metastasis, which improved due to better understanding of IMN metastasis. In the present retrospective study, 36 patients had both IMNs and distant metastases. This is likely because the output tube of IMN is located in the supraclavicular and anterior mediastinum region, entering the superior vena cava through the left and right jugular veins, thus increasing the risk of widely systemic metastases. Moreover, the metastatic rate of the extensive internal mammary lymph node exceeded 41.2%, regardless of the tumor location, molecular subtype, or tumor stage. IMNs cannot be removed during radical surgery or modified during radical mastectomy due to the abandon of the extended radical resection. As a result, active irradiation of the internal mammary lymphatic chain may reduce the risk of metastasis and recurrence of IMN, and improve the prognosis for breast cancer patients. Nevertheless, positive systemic therapy retains its important role in breast cancer treatment.

Irradiation of IMNs is problematic because it might increase the cardiac dose, as well as the risk of morbidity and mortality. Recent advances in radiotherapy technology include sophisticated imaging integrated into planning systems with techniques that shield the heart or involve deep inspiration breath holding. Such approaches allow shaping or sculpting radiation doses to suit complex cancer volumes, while reducing treatment time to protect healthy organs. These advances have the potential to further increase the added benefit of radiotherapy beyond surgery and systemic therapy, increasing survival rates.

## Conclusions

There were several limitations of this study. Firstly, this is a retrospective study, so there may exist selection bias; secondly, most of the patients did not undergo adjuvant radiotherapy owing to that most of them were treated in county hospitals before recurrence, and the others had no indications of radiotherapy according to guidelines at that time; thirdly, our study were mainly based on enhanced-CT, further studies should be done based on more accurate imageological examinations such as PET-CT and so on. In conclusion, the metastatic rate is high in the IMNs, suggesting that irradiation of the internal mammary lymphatic chain is indispensable. We propose that the upper bound of the internal mammary lymphatic chain should be the subclavian vein with a 5-mm margin, thus connecting to the caudal border of supraclavicular CTV in breast cancer patients at high risk of recurrence. Further high-quality prospective randomized trials are needed to validate this conclusion.

## Data Availability

The datasets used and/or analyzed during the current study are available from the corresponding author on reasonable request.

## Abbreviations

ALNs: Axillary Lymph Nodes
CT: Computed Tomography
CTV: Clinical Target Volume
HR: Hormone Receptor
IHC: Immunohistochemistry
IMNs: Internal Mammary Lymph Nodes
NCCN: National Comprehensive Cancer Network
PET/CT: Positron Emission Tomography/Computed Tomography
TNBC: Triple Negative Breast Cancer
